# But Does It Work? Evidence, Policy-Making and Systems Thinking Comment on "What Can Policy-Makers Get Out of Systems Thinking? Policy Partners’ Experiences of a Systems-Focused Research Collaboration in Preventive Health"

**DOI:** 10.34172/ijhpm.2020.71

**Published:** 2020-05-17

**Authors:** Tara Lamont

**Affiliations:** Wessex Institute, University of Southampton, Southampton, UK.

**Keywords:** Evidence Use, Knowledge Mobilisation, Policy-Making, Systems Thinking

## Abstract

Systems thinking provides new ways of seeing the world, focusing attention on the relationship between elements in complex systems and the spaces inbetween. Haynes study shows that many policy-makers valued systems thinking as a new way to approach old problems. But they also wanted greater focus on useful policy solutions. This raises interesting questions about the tensions between complexity and simple, action-oriented solutions and how evidence is used in decision-making. Backstage understanding of the complexity of policy problems is matched with the frontstage need to focus on what works. This reflects trends in recent public policy for evidence centres providing decision-makers with toolkits and dashboards of ‘proven’ interventions. There are good examples of evaluations using systems thinking allowing for complexity while addressing policymaker needs to be accountable for public investment and decisions. Strategic communication skills are needed to provide compelling stories which embrace systems thinking without losing clarity and impact.

Systems thinking provides a new and dynamic way of addressing wicked problems, from reducing knife crime to combating child obesity. It may be under-used as an approach in public policy. This study by Haynes et al^1^ explores in depth the views, perspectives and experiences of policy-makers engaged in system thinking on a cross-section initiative on chronic disease prevention. It provides many useful insights and ways of optimising the value of systems thinking for policy.

While the Haynes study highlights the strengths of system thinking in reframing problems of prevention, participants appeared less convinced of the ability of this approach to deliver workable solutions. Interviewees noted the value of systems thinking for policy-makers in re-setting their mental models – “*reconceptualising health problems and contexts, goals, indicators and policy solutions*.” Part of that was the process itself, policy-makers engaging actively with researchers and other stakeholders to interpret and negotiate evidence and translate this into areas of policy action. But interviewees sometimes felt overwhelmed by the complexity which was being mapped out at the expense of practical solutions.

## Complex Versus the Simple

This is nothing new. Policy scholars give many accounts of the need for public decision-makers to arrive at simple solutions which reduce uncertainties and fit existing narratives.^2^ There are real tensions for policy-makers between embracing complexity and the need for ‘hard’ impact stories. This is shown in one example, amongst many, from a recent evaluation of a pilot scheme to develop new care models in England.^3^ These were set up to provide different forms of integration – vertical (between community/primary care and specialist health providers) as well as horizontal (between health, social care, education, housing, public health and others). Checkland et al in their evaluation, using Matland’s theory of implementation success^4^ along the double axis of conflict and ambiguity, argue that, as a programme, this can be seen as low conflict given the presentation of the programme as a whole as accepted good practice in improving ‘joined-up services.’ But there was high ambiguity in how these new organisation forms were enacted. Indeed, the notion of integration itself – as an ends or a means – reflects some of this ambiguity. One of my favourite journal paper titles reflects this very well, with the header ‘If integration is the answer, what is the question?’^5^ These vanguards were given considerable freedom to define themselves. There was some deliberate fuzziness in the scope and configuration of these new organisation forms, to reflect local needs and patterns of services. Each one might have different aims and success measures, depending on what they defined as key problems. So far, so much complex systems thinking.

Yet set against this bottom-up programme, embracing complexity and local context, was the desire at the centre to show that these models ‘worked’ against a narrow set of metrics. There was a need to demonstrate impact, particularly in relation to measures such as reduced emergency hospital admissions. This was true for each individual pilot organisation and the programme as a whole. As Checkland et al stated there was “an additional purpose of ‘performing’ for an external audience – in this case, demonstrating to HM Treasury that the National Health Service (NHS) would use any additional investment wisely and that such investment would finance a change programme which would improve performance. This may also, in part at least, explain the strong emphasis in the programme on collecting and disseminating ‘good news stories’ of successful change.”^3^

## Front and Backstage

This sense of performance is interesting. Other organisational studies have drawn on interpretative policy analysis to consider the actors, staging and audience in health policy and planning. This includes a study by Shaw et al of thinktanks in the United Kingdom, contrasting the ‘front stage’ demonstration of neutrality and independence with ‘backstage’ activities of influencing and active shaping of policy agendas.^6^

There may be a sense in which the policy-makers can embrace and understand the ‘new mental models’ of systems thinking in framing and reframing issues ‘backstage.’ But they still need a ‘frontstage’ message to answer questions of return on investment and impact. This may mean reducing complex whole system interventions to single markers of effectiveness in strategic communication campaigns. The imperative is for clear demonstrations of impact. These drivers are strong – and in many ways, necessary to address issues of public accountability and value for money. There is a performative need to come up with a simple solution and to demonstrate effect in terms of lives saved or costs reduced.

## What Is Measured and What It Means

This is not just an issue about communication. There are real challenges in designing evaluations of whole-system initiatives, which are adaptive to local context and multi-faceted. These include problems of repeatability and attribution, when you cannot isolate single active ingredients or components. Carey’s review of literature on public health and systems thinking^7^ shows that more than half of published studies are commentaries and thinkpieces. There was a paucity of high quality system-wide interventions.

Hyman’s study shows repeated frustration from participants in the descriptive nature of much of this work – and this, as they note, from a self-selected set of policy-makers already engaged with a systems-led collaboration. While appreciating the real benefits of systems thinking – “*changing how they think and talk about health problems*” some also had concerns that it might “*get in the way of policy utility.*” Existing evidence is weighted towards providing rich and detailed descriptions of problems – without hard data on solutions and impact. As one participant noted “*Telling treasury and finance and ministers how complex things are is actually not that useful*.”

## Actionable Findings

The need for central policy to be informed by good evidence has led in the United Kingdom to investment in a network of What Works Centres – from the early health body set up in 1999 to provide critical appraisal of new technologies and developing clinical guidelines to more recent agencies in areas from promoting well-being to healthy ageing and stimulating local investment.^8^ Many are predicated on a rational, technocratic model with clear evidence hierarchies (privileging experimental evaluation of interventions through randomised trials and health economic assessment). Many feature standardised evidence packages for decision-makers – from crime reduction toolkits for police commissioners to educational attainment dashboards for headteachers and school governors. While each centre is different and some embrace a range of study designs and means of engaging stakeholders in developing and assuring these outputs, these centres represent a need for focused actionable findings.

Reflecting on these developments over the last twenty years, Boaz et al^9^ note how many of these activities are locked in Best and Holmes^10^ first or second stages of knowledge mobilisation – dissemination with some investment in relationships. Few achieve the third stage of establishing evidence systems, although they cite a few initiatives from the Quality Enhancement Research Initiative at the US Department of Veteran Affairs to university/health collaborations in the United Kingdom.^9^

## Impact and Insights

A simple binary of success/failure – ‘does it work?’ – is not always helpful for decision-makers. They also need to know the conditions in which interventions may work best or which parts can be adapted usefully. For instance, a study of virtual wards showed no effect in reducing emergency admissions overall.^11^ But there were useful insights about features associated with better performance, from using ward clerks to shared group practice configurations. Realist techniques foreground the learning from different perspectives, as seen in the evaluation of a large-scale service transformation modernising stroke, kidney and sexual health services in London.^12^ Together with rich insights on changes to working practice and patient pathways the study generated practical take-home lessons for service leaders on broad mechanisms and contexts associated with better outcomes.

Decision-makers may need both summative and formative evaluation^13^ – did the programme achieve its primary objectives as well as what we learned along the way. Practical guidance has been developed for those evaluating public health interventions using systems thinking which suggests replacing the focus on primary outcomes with multiple measures of change and impact. In an example on reducing health inequalities it shows how this approach might identify points in the system where interventions might best be targeted and ways of capturing measures which are meaningful to different stakeholders.^14^

There may be particular difficulties for policy-makers embracing complexity thinking in ‘top-down’ public management systems like the United Kingdom. While systems thinking demands programmes which adapt and respond to local needs, this sits uneasily with cultures of closely managed performance through targets, indicators and incentives. Matthews notes how the rhetoric of localism in recent UK governments plays against a highly centralised governing culture with a need to keep (and demonstrate) a tight grip on performance and activity. In her study of policy-making over the last ten years, she notes “despite various promises to ‘let go,’ successive governments have instead sought to ‘hold on’ to the detail of delivery.” Ministers and policy-makers may wish to delegate authority to local agents, particularly for wicked issues which may prove stubbornly resistant to intervention, while needing to demonstrate control.^15^

## Conclusions

The tensions highlighted by Haynes et al are not always irreconcilable. It is reasonable for policy-makers to want clear demonstrations of value and impact, while recognising the complex nature of public health problems and the multi-faceted programmes of interventions to address them. Cairney and Oliver have argued in an article on evidence-based policy-making that researchers need to combine scientific rigour with ‘persuasion to translate complex evidence into simple stories.’^16^ Indeed, Holmes and Best argue that strategic communication skills are undervalued in knowledge-to-action.^17^ There will always be a need – which includes a rhetorical or performative drive – to demonstrate success in ways that are meaningful in the policy and practice worlds, while understanding the wider context and influences shaping those outcomes. The imperative now is for more high quality evaluations of complex interventions driven by systems thinking and greater capacity to provide compelling narratives for and with policy-makers that are true to the science but deliver clear messages.

## Acknowledgement

With thanks to the anonymous reviewer, who highlighted the relevance of the work of Matthews on centralised forms of UK policy-making.

## Ethical issues

Not applicable.

## Competing interests

Author declares that she has no competing interests.

## Author’s contribution

TL is the single author of the paper.
